# Effect of age and sex on prescriptions for outpatients with bipolar disorder in the MUSUBI study: a cross‑sectional study

**DOI:** 10.1186/s12991-022-00415-0

**Published:** 2022-09-12

**Authors:** Yasushi Kawamata, Norio Yasui-Furukori, Naoto Adachi, Hitoshi Ueda, Seiji Hongo, Takaharu Azekawa, Yukihisa Kubota, Eiichi Katsumoto, Koji Edagawa, Eiichiro Goto, Kazuhira Miki, Masaki Kato, Atsuo Nakagawa, Toshiaki Kikuchi, Takashi Tsuboi, Reiji Yoshimura, Kazutaka Shimoda, Koichiro Watanabe

**Affiliations:** 1 Department of Psychiatry, Dokkyo Medical University School of Medicine, 880 Kitakobayashi, Mibu, Tochigi, 321-0293 Japan; 2 The Japanese Association of Neuro-Psychiatric Clinics, Kodaira, Japan; 3 Department of Neuropsychiatry, Kansai Medical University, Osaka, Japan; 4 Department of Neuropsychiatry, Keio University School of Medicine, Minato city, Japan; 5 Department of Neuropsychiatry, Kyorin University School of Medicine, Mitaka, Japan; 6 Department of Psychiatry, University of Occupational and Environmental Health, Kitakyushu, Japan

**Keywords:** Valproate, Mood stabilizer, Childbearing age, Elderly, Real world, Outpatients

## Abstract

**Background:**

Childbearing-aged female patients and elderly patients with bipolar disorder need special attention for pharmacological treatments, but current guidelines provide little information on their pharmacological treatment. In particular, the risk/benefit balance of pharmacological treatment for childbearing-aged females with bipolar disorder is a growing concern. Therefore, we aimed to address the effect of age and sex on psychotropic drug prescription for outpatients with bipolar disorder.

**Methods:**

The MUlticenter treatment SUrvey for BIpolar disorder in Japanese psychiatric clinics (MUSUBI) study was conducted, and data on age, sex, and details of pharmacological treatment were collected.

**Results:**

A total of 3106 outpatients were included in this study. Among young females (age  ≤ 39), 25% were prescribed valproate. There was no significant difference in the frequency and daily dose of valproate prescription for young females among all groups. Valproate prescriptions were significantly less frequent among young males and more frequent among middle-aged males. Lithium prescriptions were significantly less frequent among young females and more frequent among older males (age  ≥ 65) and older females. Lamotrigine prescriptions were significantly more frequent among young males and young females and less frequent among older males and older females. Carbamazepine prescriptions were significantly less frequent among young males and more frequent among older males.

**Conclusions:**

Biased information about the risk and safety of valproate and lithium for young females was suggested, and further study to correct this bias is needed. Older patients were prescribed lithium more commonly than lamotrigine. Further studies are needed to determine the actual pharmacotherapy for elderly individuals.

**Supplementary Information:**

The online version contains supplementary material available at 10.1186/s12991-022-00415-0.

## Introduction

Bipolar disorder is a recurrent chronic disorder characterized by fluctuations in mood state and energy [1]. The prevalence rate of bipolar disorder among the world’s population is approximately 1–2% [2, 3]. Pharmacological treatments are standard care to prevent and treat fluctuations in mood state. There have been several clinical practice guidelines, such as the Canadian Network for Mood and Anxiety Treatments (CANMAT 2018), Korean Medical Algorithm Project for Bipolar Disorder 2018 (KMAP-BP 2018) and the International College of Neuropsychopharmacology Treatment Guidelines for Bipolar Disorder in adults (CINP-BD-2017) [4–6].

Because some mood stabilizers have teratogenic risk, childbearing-aged females need special attention for pharmacological treatment. Lithium is known as a risk factor for Ebstein’s anomaly [7]. However, one meta-analysis reported no significantly increased risk of congenital malformations [8]. Valproate increases the risks of autism and lowers intelligence quotient (IQ) scores [9, 10]. A lower IQ score was dose-dependently more severe among children with in utero exposure to valproate by their mothers with epilepsy [9]. Carbamazepine increases the risks of spina bifida [11]. Elderly patients also need special attention for pharmacological treatment because of their lower tolerability. To prevent lithium toxicity due to a decrease in renal function, careful monitoring of serum lithium concentrations is needed [12]. Because of these issues, pharmacological treatments tend to be adjusted by age and sex.

Recent Japanese expert consensus guidelines reported that there were no first-line treatments for depressive and manic episodes in elderly individuals [13]. CANMAT 2018 reported that monotherapy with lithium or valproate was recommended as a first-line treatment for elderly individuals with acute mania. Regarding elderly individuals with depression, monotherapy with quetiapine or lurasidone was recommended as a first-line option [6]. Regarding mania in elderly individuals, KMAP-BP 2018 reported that monotherapy with atypical antipsychotics or mood stabilizers was the first-line treatment. In addition, first-line mood stabilizers included valproate and lithium, and first-line atypical antipsychotics included aripiprazole, quetiapine and olanzapine. For depression in elderly individuals, they reported that monotherapy of atypical antipsychotics or mood stabilizers, mood stabilizers plus atypical antipsychotics, atypical antipsychotics plus lamotrigine, and mood stabilizers plus lamotrigine were first-line treatments. In addition, first-line mood stabilizers included lamotrigine, valproate and lithium, and first-line atypical antipsychotics included aripiprazole, quetiapine and olanzapine [5]. For pregnant females, CANMAT 2018 reported no specific recommendation, but that valproate should be avoided [6]. KMAP-BP 2018 reported no first-line recommendation for females of childbearing age [5]. CINP-BD-2017 reported that monotherapy of atypical antipsychotics and lamotrigine (in doses  < 200 mg/day) are reasonable choices for pregnant females who need pharmacological treatments. In addition, they reported that valproate and carbamazepine should be avoided in any pregnant cases [4].

Approximately 90% of patients with bipolar disorder in Japan receive outpatient treatment, and half of them are treated at clinics that are members of Japanese Association of Neuro-Psychiatric Clinics [14]. Current guidelines provide little information on pharmacological treatment for special populations, such as childbearing-aged female and elderly patients, because they lack information on the first-line treatment or optimal dose. Therefore, we aimed to address the effect of age and sex on psychotropic drug prescription for outpatients with bipolar disorder.

### Methods

#### Participants and methods

This study (the MUlticenter treatment SUrvey on Bipolar disorder in Japanese psychiatric clinics, MUSUBI) was a cross-sectional study conducted at 176 outpatient clinics belonging to the Japanese Association of Neuro-Psychiatric Clinics [15–24]. MUSUBI was conducted between September 2016 and October 2016, and a questionnaire was administered. Patients diagnosed with bipolar disorder according to the International Classification of Diseases, tenth revision were included in this study. Clinical psychiatrists were asked to complete a questionnaire about patients, and the medical records of the patients were reviewed to obtain medication and demographic data (age and sex). We mailed 20 copies of the questionnaire to each outpatient clinic. A chlorpromazine equivalent for antipsychotics, imipramine equivalent for antidepressants and diazepam equivalent for benzodiazepine receptor agonists (BZRAs) were used for the purpose of comparison [25].

#### Statistical analysis

The data are presented as the mean and standard deviation (SD) and number (%). Those younger than 39 years were grouped into the “young” group, those 40–64 years old were grouped into the “middle-aged” group, and those older than 65 years were grouped into the “older” group. Chi-square tests were performed for categorical variables, and residual analysis was performed to compare more than three groups. Two-tailed t tests were performed for continuous variables. Dose differences in mood stabilizers among age groups or among age and sex groups were evaluated using analysis of variance (ANOVA) followed by Bonferroni correction. A value of *p * < 0.05 was considered significant. The data were analyzed using SPSS statistics software for Windows version 27.0.0.0 (Japan IBM, Tokyo, Japan) and GraphPad Prism version 9.3.1 (GraphPad Software Inc, San Diego California, USA).

### Results

Completed questionnaires of 3213 outpatients from 176 originally solicited outpatient clinics were returned. A total of 107 outpatients with missing data were excluded, which included the data lacking age (*n* = 28), sex (*n* = 3) and all blank paper (*n* = 76); thus, a total of 3106 outpatients were included in this study. The frequency of prescriptions for mood stabilizers, antipsychotics, antidepressants and BZRAs divided by age and sex is shown in Table 1. The proportion of patients who were prescribed lithium was 51.0% among males and 44.5% among females. The Chi-square test showed significant differences (*p* < 0.001). The proportion of patients who were prescribed BZRAs was significantly different between sexes (*p* = 0.015). Among the three age groups, the proportions of patients who were prescribed lithium, valproate, lamotrigine, antipsychotics, antidepressants and BZRAs were significantly different (*p* < 0.001, *p* = 0.004, *p* < 0.001, *p* < 0.001, *p* = 0.002, *p* < 0.001, respectively). Residual analysis revealed significant differences in the young group and older group among those prescribed lithium (Additional file 1: Table S1). Regarding valproate, the young group and middle-aged group were significantly different. Regarding lamotrigine, the young group and older group were significantly different. Regarding antipsychotics, the middle-aged group and older group were significantly different. Regarding antidepressants, the middle-aged group and older group were significantly different. Regarding BZRAs, the young group and middle-aged group were significantly different.

**Table 1:** Numbers (%) of drugs for mood stabilizers, antipsychotics, antidepressants and BZRAs divided by age and sex

	Sex			Age (years)			
	Males	Females	Significance	≤ 39	40–64	≥ 65	Significance
	*N* = 1408	*N* = 1698		*N* = 676	*N* = 1899	*N* = 531	
Mood stabilizers							
Lithium	718 (51.0%)	755 (44.5%)	< 0.001	287 (42.5%)	902 (47.5%)	284 (53.5%)	< 0.001
Valproate	408 (29.0%)	474 (27.9%)	0.513	160 (23.7%)	575 (30.3%)	147 (27.7%)	0.004
Lamotrigine	337 (23.9%)	397 (23.4%)	0.717	200 (29.6%)	465 (24.5%)	69 (13.0%)	< 0.001
Carbamazepine	72 (5.1%)	77 (4.5%)	0.452	21 (3.1%)	103 (5.4%)	25 (4.7%)	0.053
Other medications							
Antipsychotics	777 (55.1%)	911 (53.6%)	0.393	387 (57.2%)	1075 (56.6%)	226 (42.5%)	< 0.001
Antidepressants	566 (40.1%)	713 (41.9%)	0.312	271 (40.0%)	822 (43.2%)	186 (35.0%)	0.002
BZRAs	969 (68.8%)	1236 (72.7%)	0.015	443 (65.5%)	1391 (73.2%)	371 (69.8%)	< 0.001

*BZRAs*, benzodiazepine receptor agonists

The daily doses of mood stabilizers, antipsychotics, antidepressants and BZRAs divided by age and sex are shown in Table 2. The daily dose of lithium was significantly higher among males than females (*p* < 0.001). Among the three aged groups, the doses of lithium, valproate, antipsychotics, and antidepressants were significantly different by one-way ANOVA (*p* < 0.001, *p* < 0.001, *p* = 0.018, *p* = 0.024, respectively). Bonferroni’s post hoc analysis showed that the daily dose of lithium in the older group was significantly lower than that in the young group and middle-aged group (*p* < 0.001, *p* < 0.001, respectively). Regarding valproate, the daily dose in the middle-aged group was significantly higher than that in the older group (*p* < 0.001). Regarding antipsychotics, the daily dose in the middle-aged group was significantly higher than that in the older group (*p* = 0.047). Regarding antidepressants, the daily dose in the middle-aged group was significantly higher than that in the older group (*p* = 0.027).

**Table 2:** Dose of drugs for mood stabilizers, antipsychotics, antidepressants and BZRAs divided by age and sex

	Sex			Age (years)			
	Males	Females	Significance	≤ 39	40–64	≥ 65	Significance
	*N* = 1408	*N* = 1698		*N* = 676	*N* = 1899	*N* = 531	
Mood stabilizers							
Lithium	599.9 (248.1)	519.2 (235.4)	< 0.001	601.7 (275.7)	579.7 (241.0)	447.5 (186.0)	< 0.001
Valproate	535.8 (272.0)	504.2 (268.9)	0.083	490.6 (281.6)	543.7 (273.4)	451.9 (231.6)	< 0.001
Lamotrigine	160.2 (98.6)	158.2 (103.0)	0.780	159.9 (98.4)	161.1 (103.6)	143.0 (88.5)	0.376
Carbamazepine	354.9 (208.8)	372.1 (197.4)	0.606	352.3 (199.0)	384.4 (211.7)	288.0 (145.9)	0.098
Other medications							
Antipsychotics	206.0 (227.4)	218.8 (264.1)	0.302	196.2 (209.5)	225.9 (268.5)	180.3 (201.4)	0.018
Antidepressants	125.2 (101.1)	127.1 (106.3)	0.746	121.9 (97.6)	131.5 (104.7)	109.4 (108.1)	0.024
BZRAs	14.4 (19.5)	15.1 (28.1)	0.484	14.3 (27.4)	15.6 (25.0)	12.4 (19.9)	0.078

*BZRAs*, benzodiazepine receptor agonists

Table 3 shows the five most often prescribed drugs for antipsychotics, antidepressants and BZRAs divided by age and sex. Regarding antipsychotics, aripiprazole was the most often prescribed antipsychotic among young males (25.7%), young females (26.4%), middle-aged males (20.2%) and middle-aged females (20.6%). Olanzapine was the most often prescribed antipsychotic among older males (12.4%), and quetiapine was the most often prescribed antipsychotic among older females (11.4%). Regarding antidepressants, duloxetine was the most often prescribed antidepressant among middle-aged males (8.6%), middle-aged females (7.9%) and older females (7.1%). Escitalopram was the most often prescribed antidepressant among young males (9.5%) and young females (8.7%), and mirtazapine was the most often prescribed antidepressant among older males (7.6%). Flunitrazepam was the most often prescribed BZRA among young males (12.6%), young females (13.6%), middle-aged males (21.7%), middle-aged females (22.2%), older males (21.5%) and older females (21.1%).

**Table 3:** Numbers (%) and dose of the five most often prescribed drugs divided by age and sex

	*n* (%)	Mean dose (SD)		*n* (%)	Mean dose (SD)
Antipsychotics					
Young males			Young females		
Aripiprazole	65 (25.7)	5.4 (5.6)	Aripiprazole	112 (26.4)	7.5 (7.0)
Olanzapine	42 (16.6)	5.3 (4.4)	Quetiapine	52 (12.2)	129.6 (163.0)
Quetiapine	29 (11.5)	80.5 (74.7)	Olanzapine	50 (11.7)	5.1 (4.6)
Sulpiride	15 (5.9)	100.0 (53.4)	Risperidone	20 (4.7)	1.4 (1.0)
Levomepromazine	9 (3.5)	12.3 (15.1)	Levomepromazine	19 (4.4)	32.3 (37.4)
Middle-aged males			Middle-aged females		
Aripiprazole	192 (20.2)	7.2 (6.9)	Aripiprazole	197 (20.6)	8.1 (7.7)
Olanzapine	126 (13.3)	6.2 (5.1)	Olanzapine	137 (14.3)	6.7 (5.4)
Quetiapine	104 (10.9)	149.0 (157.5)	Quetiapine	126 (13.2)	137.3 (174.3)
Sulpiride	73 (7.7)	122.9 (69.8)	Levomepromazine	57 (5.9)	30.5 (55.6)
Levomepromazine	50 (5.2)	26.1 (29.8)	Risperidone	41 (4.3)	1.8 (1.3)
Older males			Older females		
Olanzapine	26 (12.4)	5.8 (3.5)	Quetiapine	37 (11.4)	88.3 (113.1)
Quetiapine	20 (9.5)	110.0 (158.7)	Olanzapine	35 (10.8)	7.1 (5.7)
Levomepromazine	13 (6.2)	26.1 (25.0)	Aripiprazole	24 (7.4)	6.8 (5.9)
Sulpiride	11 (5.2)	103.8 (65.1)	Levomepromazine	15 (4.6)	13.2 (8.8)
Aripiprazole	8 (3.8)	13.8 (10.8)	Sulpiride	12 (3.7)	97.9 (45.7)
Antidepressants					
Young males			Young females		
Escitalopram	24 (9.5)	13.3 (7.6)	Escitalopram	37 (8.7)	12.4 (5.3)
Duloxetine	23 (9.1)	36.0 (16.1)	Duloxetine	37 (8.7)	37.8 (17.8)
Mirtazapine	14 (5.5)	23.0 (11.9)	Sertraline	29 (6.8)	57.3 (30.5)
Paroxetine	11 (4.3)	21.1 (11.3)	Trazodone	27 (6.3)	46.2 (38.5)
Sertraline	6 (2.3)	62.5 (30.6)	Paroxetine	22 (5.1)	24.3 (15.4)
Middle-aged males			Middle-aged females		
Duloxetine	82 (8.6)	38.7 (16.4)	Duloxetine	76 (7.9)	42.6 (16.1)
Mirtazapine	80 (8.4)	22.5 (12.9)	Escitalopram	63 (6.6)	12.9 (5.6)
Trazodone	57 (6.0)	49.0 (33.2)	Mirtazapine	63 (6.6)	26.7 (12.2)
Paroxetine	51 (5.3)	22.1 (13.2)	Trazodone	58 (6.0)	46.9 (26.1)
Escitalopram	46 (4.8)	12.6 (5.2)	Paroxetine	46 (4.8)	22.9 (13.7)
Older males			Older females		
Mirtazapine	16 (7.6)	23.9 (9.1)	Duloxetine	23 (7.1)	41.7 (14.9)
Trazodone	9 (4.3)	55.5 (24.2)	Mirtazapine	22 (6.8)	19.4 (14.5)
Duloxetine	8 (3.8)	43.7 (15.0)	Fluvoxamine	16 (4.9)	68.7 (40.5)
Amoxapine	7 (3.3)	51.4 (30.3)	Trazodone	14 (4.3)	35.7 (12.8)
Fluvoxamine	7 (3.3)	75.0 (38.1)	Paroxetine	13 (4.0)	12.3 (12.1)
BZRAs					
Young males			Young females		
Flunitrazepam	32 (12.6)	1.8 (0.8)	Flunitrazepam	58 (13.6)	2.1 (1.1)
Zolpidem	27 (10.7)	8.7 (2.2)	Brotizolam	50 (11.7)	0.3 (0.6)
Brotizolam	26 (10.3)	0.2 (0.1)	Etizolam	40 (9.4)	1.4 (1.6)
Triazolam	24 (9.5)	0.3 (0.1)	Alprazolam	38 (8.9)	0.9 (0.4)
Etizolam	19 (7.5)	1.2 (0.8)	Zolpidem	37 (8.7)	9.1 (2.0)
Middle-aged males			Middle-aged females		
Flunitrazepam	206 (21.7)	1.9 (1.7)	Flunitrazepam	212 (22.2)	1.9 (0.9)
Brotizolam	131 (13.8)	0.2 (0.1)	Brotizolam	150 (15.7)	0.4 (2.0)
Triazolam	90 (9.5)	0.4 (1.0)	Zolpidem	90 (9.4)	9.5 (9.9)
Etizolam	86 (9.0)	1.4 (0.9)	Triazolam	88 (9.2)	0.3 (0.1)
Zolpidem	75 (7.9)	9.0 (2.0)	Etizolam	86 (9.0)	1.4 (1.7)
Older males			Older females		
Flunitrazepam	45 (21.5)	1.5 (0.6)	Flunitrazepam	68 (21.1)	1.8 (0.7)
Brotizolam	28 (13.3)	0.6 (1.8)	Brotizolam	51 (15.8)	0.2 (0.1)
Triazolam	18 (8.6)	0.2 (0.1)	Etizolam	29 (9.0)	0.9 (0.6)
Etizolam	17 (8.1)	1.2 (0.7)	Zolpidem	23 (7.1)	7.2 (2.7)
Zolpidem	15 (7.1)	9.0 (3.8)	Alprazolam	18 (5.5)	0.8 (0.5)

*BZRAs*, benzodiazepine receptor agonists

**Fig. 1:**
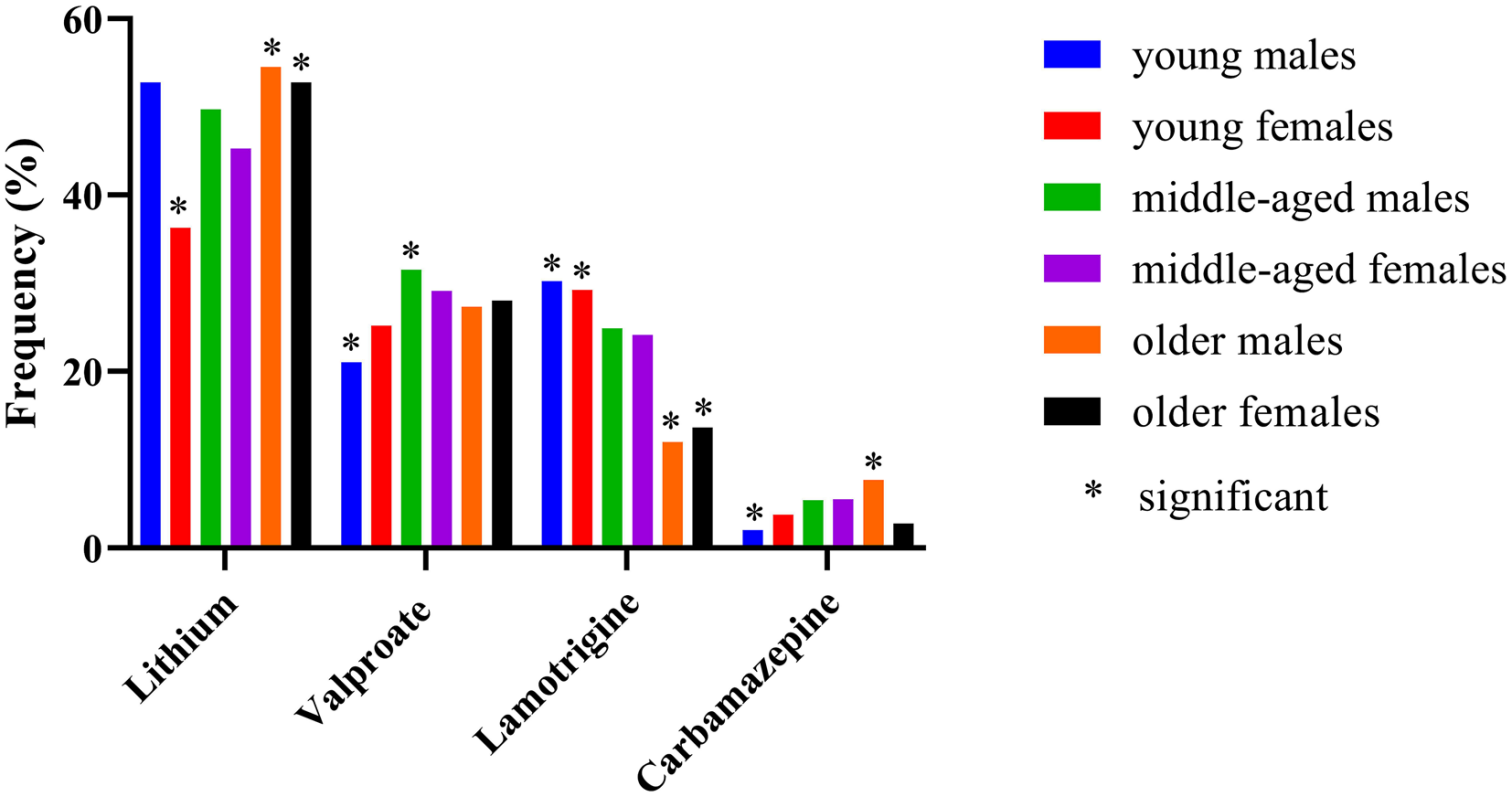
Prescriptions of mood stabilizers by age and sex. *p < 0.05

The comparison of frequencies of mood stabilizer prescriptions in each age–sex group is shown in Fig. 1. Significant differences in the frequencies of lithium, valproate, lamotrigine and carbamazepine were shown by the Chi-square test (*p* < 0.001, *p* = 0.018, *p* < 0.001, *p* = 0.019, respectively). Residual analysis showed significant differences in the frequency of lithium prescriptions among young females, older males and older females (Additional file 2: Table S2). The frequencies of valproate prescriptions among young males and middle-aged males were significantly different. The frequencies of lamotrigine prescriptions among young males, young females, older males and older females were significantly different. The frequencies of carbamazepine prescriptions among young males and older males were significantly different.

**Fig. 2:**
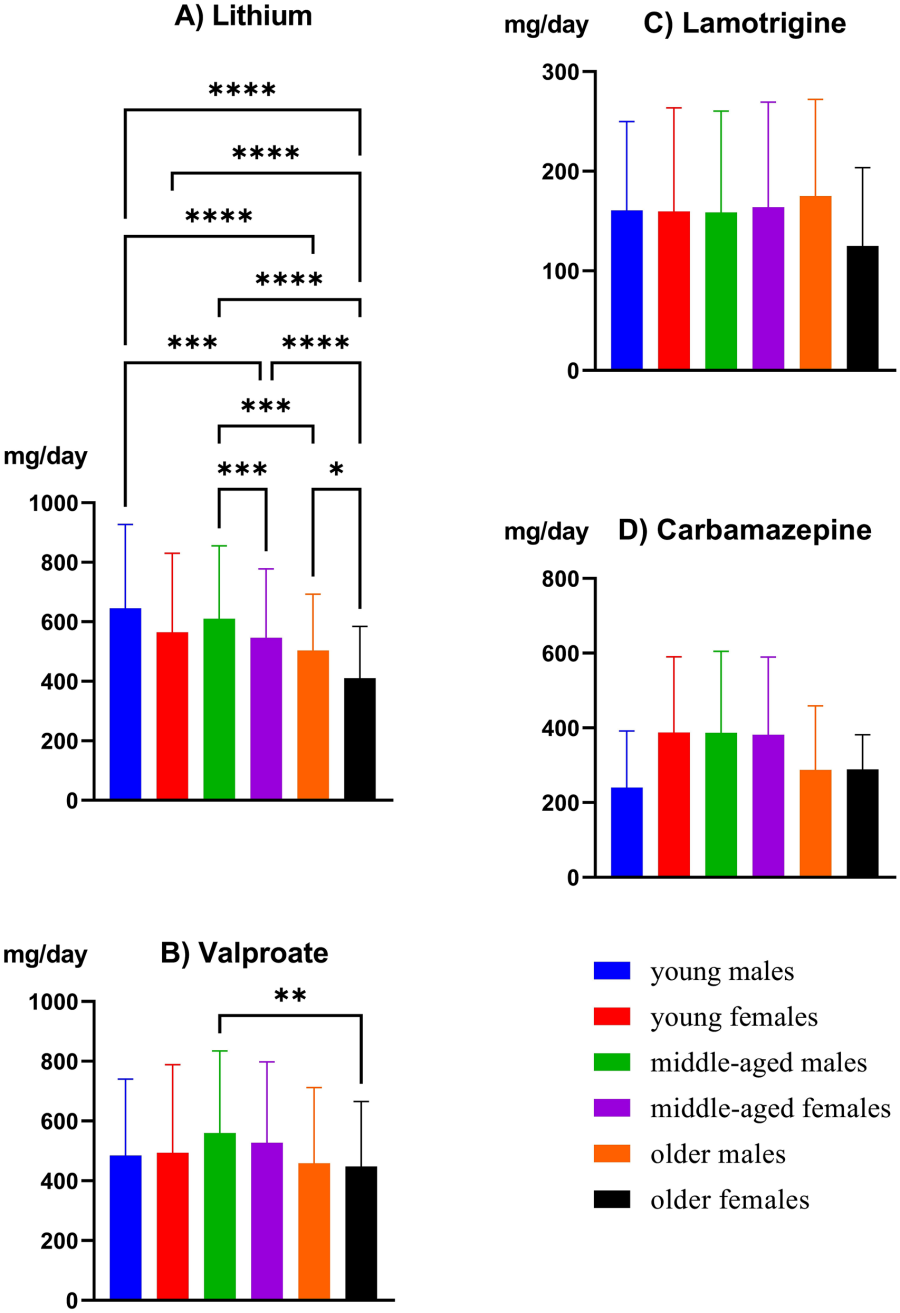
Daily dose of mood stabilizers by age and sex. *p < 0.05, **p < 0.01, ***p < 0.001, ****p < 0.0001

The comparison of daily doses for mood stabilizer prescriptions in each age–sex group is shown in Fig. 2. Significant differences were shown by one-way ANOVA regarding lithium and valproate (*p* < 0.001, *p* = 0.003, respectively). Bonferroni’s post hoc analysis showed that the daily dose of lithium among young males was significantly higher than that among middle-aged females, older males and older females (*p* < 0.001, *p* < 0.0001, *p* < 0.0001, respectively). The daily dose of lithium among young females was significantly higher than that among older females (*p* < 0.0001). The daily dose of lithium among middle-aged males was significantly higher than that among middle-aged females, older males and older females (*p* < 0.001, *p* < 0.001, *p* < 0.0001, respectively). The daily dose of lithium among middle-aged females was significantly higher than that among older females (*p* < 0.001). The daily dose of lithium among older males was significantly higher than that among older females (*p* = 0.016). Regarding valproate, the daily dose among middle-aged males was significantly higher than that among older males (*p* = 0.008).

## Discussion

This study had two main findings. First, among young females in our survey, 25% were prescribed valproate. In addition, there was no significant difference in the frequency and daily dose of valproate prescription for young females among all groups. In contrast, lithium prescription for young females was significantly rare. Second, among older males and older females, lithium prescription was significantly frequent, and lamotrigine prescription was significantly rare. To the best of our knowledge, this is the first study to report psychotropic drug prescriptions, including information about the frequency and daily dose, for childbearing-aged female and elderly outpatients with bipolar disorder in Japan.

Lithium was significantly less commonly prescribed to young women in this study. This is partly because psychiatrists are concerned about the risk of Ebstein's malformation [7] with lithium and because lithium is contraindicated in pregnant and parturient women in Japan. Regarding lithium, an overestimated risk of congenital malformation has been reported [26]. The risk/benefit balance of lithium for pregnant females has been debated [8]. For females of childbearing age, on the other hand, the risk of neurodevelopmental disabilities caused by valproate has become a growing concern. In addition, a worldwide survey reported that 41% of pregnancies were unintended [27]. Therefore, some guidelines have suggested against prescribing valproate for females of childbearing age [4, 6]. Valproate is contraindicated in Japan in principle for pregnant and parturient women. Nevertheless, there was no significant discrepancy in the frequency of valproate prescriptions between young women and other groups in this study. This result may be supported by a previous study [28] that surveyed Japanese psychiatrists' attitudes toward prescribing valproic acid to women of childbearing age, which lacked data on actual valproic acid prescription but reported that Japanese psychiatrists often prescribe valproic acid to women of childbearing age. The previous study [28] also reported that psychiatrists working at general hospitals tended to refrain from prescribing valproate to childbearing-aged female compared to those affiliated with other medical facilities, such as psychiatric hospitals and private clinics. Little chances and experience of psychiatrists working at clinics in consulting for pregnant female with psychiatric diseases might increase their valproate prescription to women of childbearing age. This finding supports the reported awareness of the high incidence of valproic acid prescription by Japanese psychiatrists to women of childbearing age. For younger women, lamotrigine is the preferred choice because of its relative safety during pregnancy [4].

Among elderly individuals, lithium and carbamazepine were commonly prescribed for older men, while lamotrigine was rarely prescribed. This may be due to the repeated long-term prescription of lithium and carbamazepine, which are older drugs than lamotrigine. This may be a reasonable result, since drugs that are effective in adults are also effective in older bipolar patients [29, 30]. Because lithium prescriptions are declining worldwide [31–34], older patients who are repeatedly prescribed lithium on a long-term basis are more likely than younger or middle-aged patients to be prescribed lithium more frequently. Similarly, given the downward trend in carbamazepine prescriptions [33], older men who are repeatedly prescribed carbamazepine on a long-term basis may have a statistically increased frequency of carbamazepine prescription compared to younger men. Furthermore, valproic acid prescriptions were significantly less frequent among younger men and more frequent among middle-aged men. However, there is no clear explanation as to why this significant difference occurs only among men. Since lamotrigine prescriptions are on the rise worldwide [32, 34], it is possible that younger patients, likely new patients, were prescribed lamotrigine more frequently, while older patients on ongoing treatment were repeatedly prescribed the older mood stabilizer. There is no clear explanation for this significant discrepancy. Due to the lack of data on the duration of illness in this study, it is unclear whether the prescription data in this study started with elderly individuals. Further studies are needed to determine the actual pharmacotherapy for elderly individuals.

The daily dose of valproic acid in elderly women was significantly lower than that in middle-aged men. This may be due to the higher proportion of free valproic acid in the serum of elderly women. Approximately 90% of valproic acid is protein bound, and women and elderly individuals tend to have relatively high free valproic acid concentrations [35]. Regarding the daily dose of lithium, the present study showed that it was significantly reduced by older age and female sex. This result seems reasonable since lithium is excreted by the kidneys and creatinine clearance decreases with age and in women [12].

Regarding antipsychotics, prescription frequency was significantly rare in the older group and significantly frequent in the young group. This might be due to the upward trend in prescribing antipsychotics [31–33]. The daily dose of antipsychotics was significantly lower in the older group than in the middle-aged group. This is reasonable because of elderly patients’ lower tolerability to antipsychotics [36]. Aripiprazole, olanzapine, and quetiapine were the most often prescribed antipsychotics in our survey. A previous study in Denmark reported that aripiprazole, olanzapine, and quetiapine were the most often prescribed antipsychotics for patients with bipolar disorder [32], and our findings were similar to the results of that previous study. Lurasidone was not listed because this drug was approved in 2020 in Japan. Regarding antidepressants in our findings, the frequency of prescription was significantly rare in the older group and frequent in the middle-aged group. In addition, the daily dose of antidepressants in the older group was significantly lower than that in the middle-aged group. We do not have a clear explanation for this significant difference. This might be due to the greater risk of adverse events in elderly individuals because of multiple medical comorbidities and drug–drug interactions in the case of polypharmacy [37]. In our survey, escitalopram, duloxetine, and mirtazapine were the most often prescribed antidepressants. In some guidelines, the efficacy of adjuvant selective serotonin reuptake inhibitor (SSRI) therapy was reported [6, 38], but the efficacy of serotonin and norepinephrine reuptake inhibitor (SNRI) or mirtazapine therapy was not reported. Regarding BZRAs in our findings, the frequency of prescription was significantly frequent among females. Female sex is a known risk factor for long-term BZRA use [39], which is in line with our findings. In addition, BZRA prescriptions were significantly frequent in the middle-aged group and significantly rare in the young group. Because of the dependence and long-term use of BZRAs [40], prescription in the middle-aged group might have been relatively frequent.

There are several limitations of this study. First, because our study was a cross-sectional study, it could not elucidate causal relationships. Second, this study lacked structural clinical evaluations for patients. Third, there may be selection bias because the participants were not randomized. Fourth, our sample heterogeneously had bipolar I disorder or bipolar II disorder, which were not distinguished. Our sample was also heterogeneous in terms of current mood status, such as manic state and depressive state. Pharmacological treatment can differ according to mood status. Fifth, several potential confounding factors, such as comorbidities, willingness to become pregnant and duration of illness, were not assessed in our study. Finally, coadministration of mood stabilizers was not assessed.

## Conclusion

Among young females, 25% were prescribed valproate. There was no significant difference in the frequency and daily dose of valproate prescription for young females among all groups. Lithium prescription for young females was significantly rare. Further study to correct biased information about the risk and safety of valproate and lithium for young females is needed. Among elderly individuals, lithium prescription was significantly frequent, and lamotrigine prescription was significantly rare. Further study to search for real-world pharmacological treatments for elderly individuals is needed.

## Supplementary Information

**Additional file 1**: **Table S1** Adjusted standardized residuals of mood stabilizers, antipsychotics, antidepressants and benzodiazepine receptor agonists divided by age.

**Additional file 2**: **Table S2** Adjusted standardized residuals of mood stabilizers divided by age and sex.

## Data Availability

The ethics committee of the Japanese Association of Neuro-Psychiatric Clinics set restrictions on data sharing because the data contain potentially identifying or sensitive patient information. Please contact the institutional review board of the ethics committee when requesting data. Contact information for our ethics committee: The institutional review board of the ethics committee of the Japanese Association of Neuro-Psychiatric Clinics; Shibuya-ku, Yoyogi 1-38-2, Tokyo Metropolis, Japan, Postal Code 151–0053, Phone + 81-3-3320-1423.

## Abbreviations

CANMAT: The Canadian Network for Mood and Anxiety Treatments 2018
KMAP-BP 2018: Korean Medical Algorithm Project for Bipolar Disorder 2018
CINP-BD-2017: The International College of Neuropsychopharmacology Treatment Guidelines for Bipolar Disorder in adults
BZRAs: Benzodiazepine receptor agonists
MUSUBI: The MUlticenter treatment SUrvey on Bipolar disorder in Japanese psychiatric clinics
